# Poretti-Boltshauser Syndrome: A Report of Two Cases From Bahrain With a Novel Mutation and Literature Review

**DOI:** 10.7759/cureus.77172

**Published:** 2025-01-09

**Authors:** Zahra Alsahlawi, Ameera M Barni, Bayan M Barni, Hatem Abbod, Fatema Kadhem, Hussain Al Harmi

**Affiliations:** 1 Department of Pediatrics, Arabian Gulf University, Manama, BHR; 2 Department of Pediatrics, Salmaniya Medical Complex, Manama, BHR; 3 Department of Radiology, Salmaniya Medical Complex, Manama, BHR; 4 Department of Ophthalmology, Salmaniya Medical Complex, Manama, BHR

**Keywords:** bahrain, cerebellar cyst, high myopia, lama 1 gene, poretti-boltshauser syndrome

## Abstract

Poretti-Boltshauser syndrome (PTBHS) is a neuro-ophthalmological rare genetic disease that has an autosomal recessive inheritance that occurs as a consequence of a mutation in the LAMA1 gene. This gene is important in the development of blood vessels and certain organs, including the cerebellum and the retina. PTBHS is characterized by specific cerebellar abnormalities that manifest in the development of certain clinical features, including cerebellar ataxia, intellectual disability, and delayed language and motor development. In addition to the cerebellar manifestations, retinal abnormalities were noted in patients with PTBHS, such as ocular motor apraxia, severe myopia, strabismus, and retinal dystrophy.

In this report, we describe a three-year-old female child diagnosed with PTBHS after assessing and reviewing her clinical presentation and work-up results at Salmaniya Medical Hospital. She presented with nystagmus, poor eye fixation, and delayed gross motor development. The MRI findings were almost subtle, and the diagnosis was confirmed mainly based on the genetic results. *LAMA1*: c.1243del chr18-7042162TG>T (GRCh37 format) or p.His415Ilefs*78 with transcript ID as NM_005559.4 homozygous mutation was found by whole exome sequencing. Her older sister was also diagnosed with the same disease afterwards by target mutation.

Most genetic diseases are difficult to cure, and the management relies mainly on supportive treatment. For that, doctors should pay close attention to the small details in the clinical presentation and investigation findings and always involve a specialized doctor in case any small concern is raised.

## Introduction

Poretti-Boltshauser syndrome (PTBHS) is a rare autosomal recessive disorder caused by* LAMA1* (laminin1) (Online Mendelian Inheritance in Man (OMIM): 150320) mutation [1]. *LAMA1* gene encodes for laminin subunit alpha-1 (400-KD), which links to laminin beta and laminin gamma subunits by disulfide bonds, and this laminin trimer acts as an adhesion protein for endothelial cells and hence it plays a role in the organization of the extracellular matrix as well as the polarization in the basal and apical areas [2,3]. This protein has been involved in axonal guidance (pathfinding) and the development of blood vessels; subsequently, it can interfere with the development of certain organs, including the cerebellum and the retina [3].

PTBHS is a neuro-ophthalmological disease characterized by specific cerebellar abnormalities that are typically seen on brain imaging, including cerebellar dysplasia, cerebellar cyst, cerebellar vermis hypoplasia, and 4th ventricle enlargement that present with phenotypes that include non-progressive cerebellar ataxia, intellectual disability, and delayed language and motor development [4]. Retinal abnormalities described in PTBHS as retinal dystrophy include ocular motor apraxia, severe myopia, strabismus, lattice degeneration, and retinal pigment changes and degeneration [5].

Overall, there is a lack of research on the topic of PTBHS. A recent article published in February 2023 mentioned that the total number of case reports published was about 41 cases extending through the period from 2014 to 2022 [6]. Among the 41 cases, only one case was reported in the Arab region, which was from Saudi Arabia [6]. Hence, in this report, we discuss two patients diagnosed with this syndrome in Bahrain along with some literature review.

## Case presentation

Case 1

This is a case of a four-year-old female baby who was a product of a spontaneous full-term normal vaginal delivery to consanguineous parents and weighed 3.1 kg at birth. There were no notable perinatal complications, and she did not require admission to the neonatal intensive care unit. She was seen first in the clinic of genetic and metabolic diseases at our hospital (Salmaniya Medical Complex) at the age of two months with a history of nystagmus, weak eye fixation, and delayed motor skills. As per her parents, nystagmus started in the early few days of her life and the delayed motor skills started showing as she grew up. On neurological examination, she demonstrated some cerebellar deficits such as ataxic gait and nystagmus.

Since the main complaint was related to her vision, she was referred to the ophthalmology clinic where she had multiple regular visits. At the age of two years, a full fundus examination was done. On the right side (OD), she had severe chorioretinal degeneration, white without pressure (WWP), dull macular reflex, and pale disc. While on the left side (OS), she had the same findings except that one dot of retinal pigment epithelium (RPE) mottling was detected. At this age, her eyeglasses prescription was as follows: right: -14.5; Dsph (diopter sphere): -2.0; Dcyl: (cylindrical diopter) 80; left: -13.5; Dsph: -2.5; Dcyl: 75. Through these follow-ups and continuous visits, where the examination is done and the prescription of the eyeglasses changed accordingly, the patient by this age had improved much and had started paying attention to moving objects and light.

**Figure 1:**
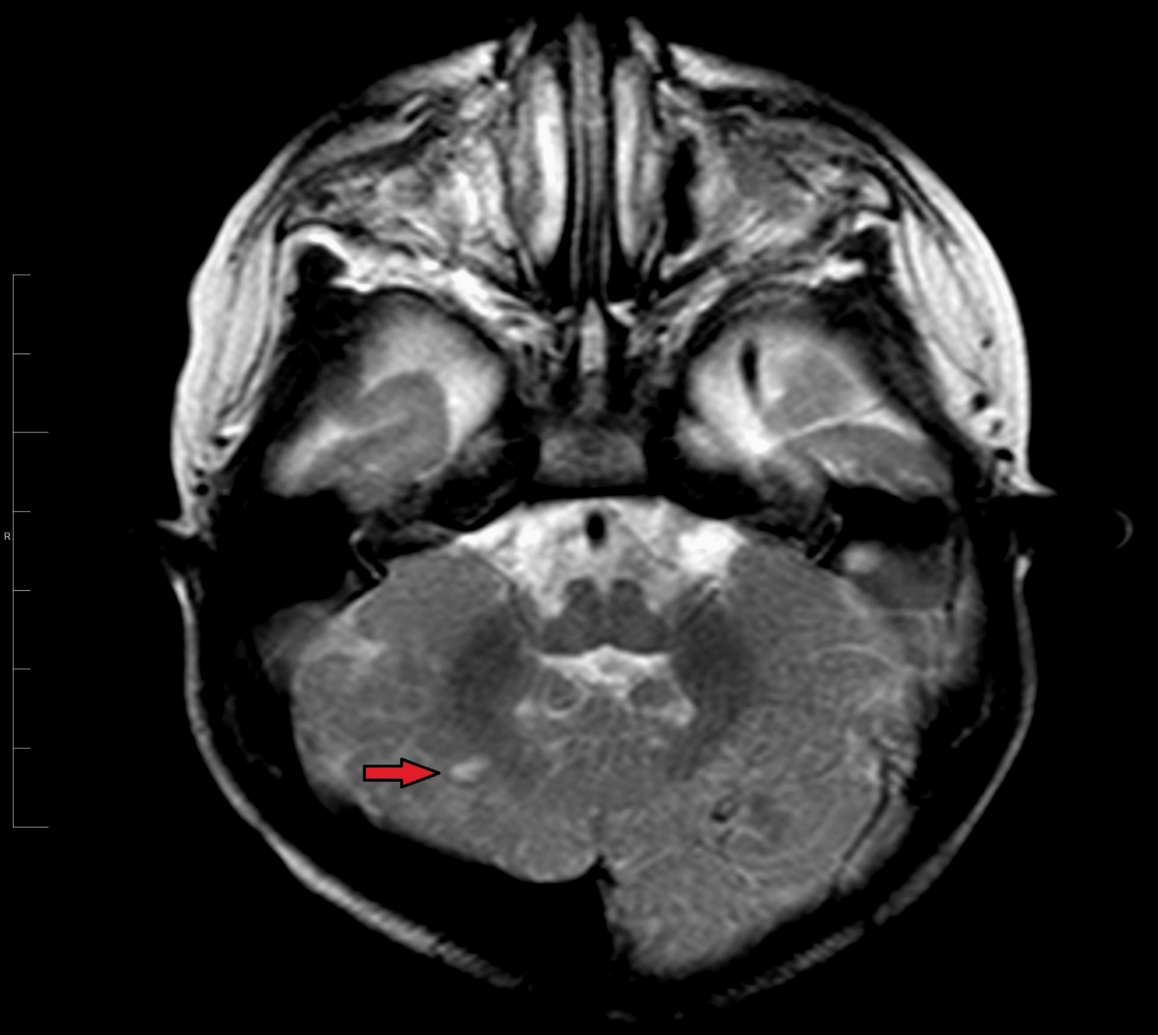
An axial section of the T2 brain MRI scan of case 1 showing abnormal cerebellar foliation and a right parenchymal cerebellar cyst. The red arrow is pointing toward the mentioned parenchymal cerebellar cyst.

**Figure 2:**
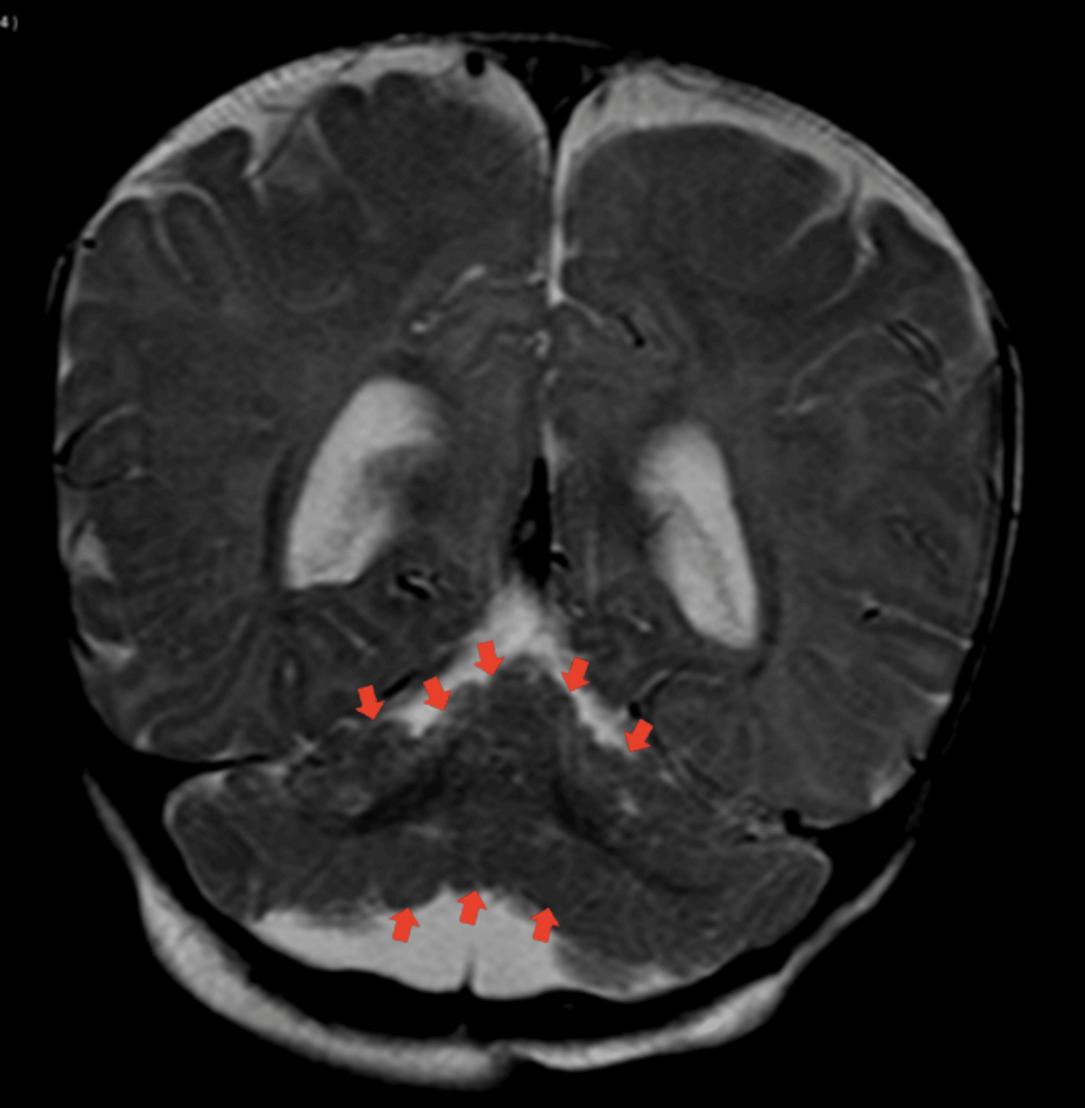
A coronal section of the T2 brain MRI scan of case 1 showing abnormal cerebellar foliation as labeled with the red arrows.

As there were clear neurological manifestations, an MRI scan of the brain was done. It was very difficult to tell if there were any striking abnormal findings if the radiologist relied on the pictures alone without knowing the background history of the patient. As this case was discussed in more depth with our consultant radiologist, much attention was paid to the small details of the imaging, and some changes were detected, including abnormal cerebellar foliation and a parenchymal cerebellar cyst. Below you can find two brain MRI images of this patient illustrating the changes (Figures 1, 2).

Interestingly, this case has an older female sibling who exhibited the same neuro-ophthalmological manifestations, which prompted the physician to consider the possibility of a genetic problem.

Case 2

This is a six-year-old girl who was referred to the genetic and metabolic clinic with a very similar clinical history as her younger sister; however, since this has happened before, it was more shocking for the parents. As in case 1, no concerns were noted antenatally, but when she was only a few days old, the parents noticed the nystagmus (tremor of her pupils, as the parent described), which was associated with weak eye fixation. This continued even after the six months of her life. At the local health center, a cerebellar examination was done for the child, and she was found to have some deficits that required them to follow up with a pediatrician. At the age of four months, the parents visited a pediatrician in another secondary care hospital, and he advised them to do a brain MRI. When the imaging result came out, the parents were told that there were some changes in the cerebellum, which are suggestive of Dandy-Walker syndrome. Unfortunately, the patient was mistakenly diagnosed with this syndrome. It was explained to them that this syndrome is congenital and not inherited and hence there is no risk of having another child with the same condition. Nevertheless, after two years, the mother got pregnant again with the younger sibling (case 1).

**Figure 3:**
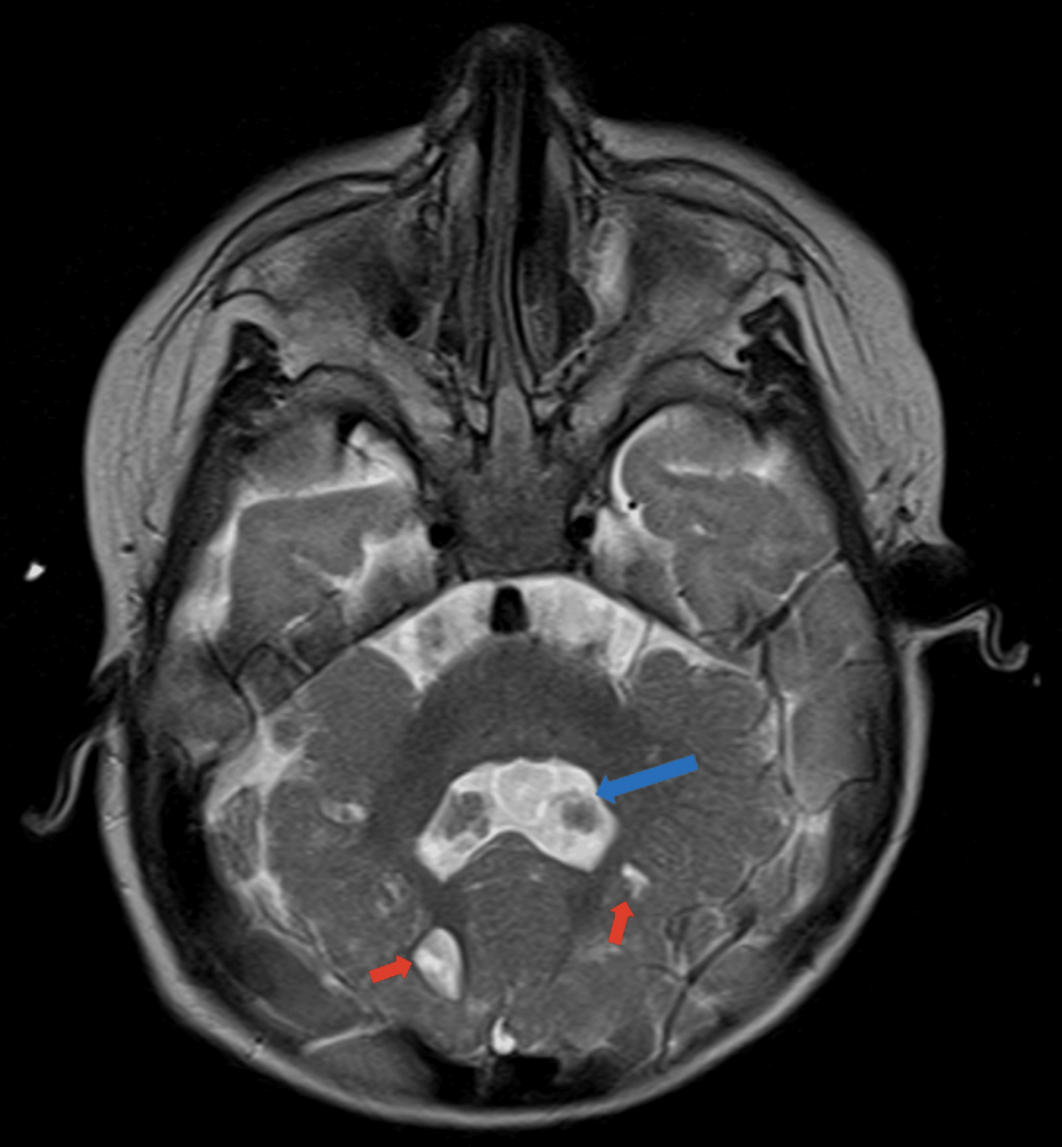
An axial section of the T2 brain MRI scan of case 2. Red arrows: cerebellar parenchymal cysts. Blue arrow: dilated fourth ventricle.

A previous brain MRI was re-reviewed in our hospital, and very similar findings to her sister were detected, including cerebellar parenchymal cysts and abnormal cerebellar foliation; however, she demonstrated an additional finding, i.e., a dilated fourth ventricle (Figure 3).

Genetic results

In the genetic clinic, a genetic test was done for both sisters, which revealed a mutation in the *LAMA1* gene* *that confirmed the diagnosis of a rare genetic disorder called Poretti-Boltshauser syndrome. To provide more details about the genetic tests, case 1 had a whole exome sequencing at Centogene Laboratory in Germany. The results for this case identified a mutation in the *LAMA1* gene, which is homozygous, variant c.1243del, with amino acid changes p. (His415llefs*78), single nucleotide polymorphism (SNP) identifier N/A, polyphen N/A, and Align-GVGD and SIFT N/A for SLICO parameters. These genetic changes create a shift in the reading frame that starts at codon 415 resulting in a new reading frame that ends in a stop codon 77 position downstream. According to ClinVar lists (variation ID: 419085), this variant is classified as likely pathogenic (class 2) based on the American College of Medical Genetics (ACMG) recommendation, which classifies this centogene variant as likely class 2 (likely pathogenic).

**Figure 4:**
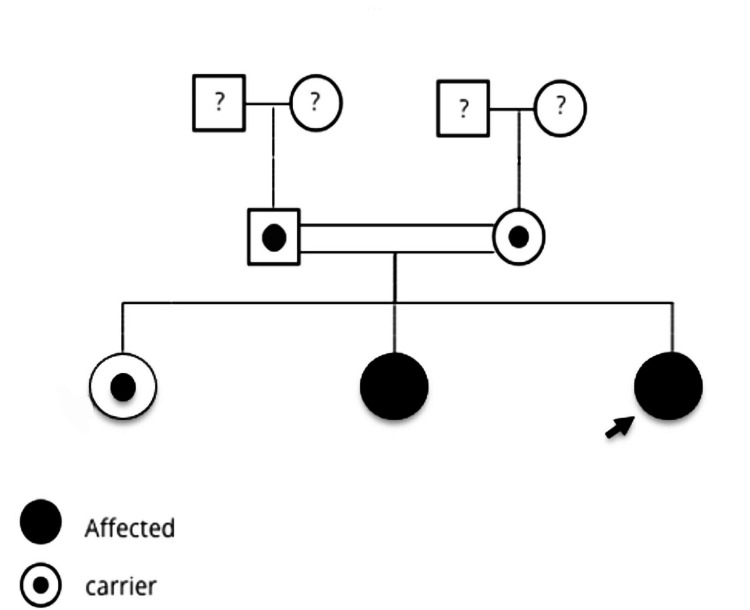
Pedigree of the patient. The parents are consanguineous and carriers of the affected gene. A girl sibling is also a carrier of the same gene but the index case and a second girl sibling are affected by the syndrome since they have inherited the two defective genes from both carrier parents.

Because of that, a genetic test was done for the rest of the members of this family, which consisted of the parents and two other daughters. This confirmed the diagnosis of the same syndrome (PTBHS) in case 2 through the presence of the same target mutation in a homozygous pattern. However, the oldest sister was a carrier of the defective gene, as were the parents (Figure 4).

Both patients were followed closely in genetic and ophthalmology clinics. There is no specific treatment for their condition except for ophthalmological support with eyeglasses, which was provided for them and showed improvement in their attention and interaction with their surroundings. They remained stable and had appropriate developmental progression according to their age with no major deterioration of their condition.

## Discussion

Most of the published case reports reviewed from the PubMed database about PTBHS focused on discussing the identified *LAMA1* gene mutation, the typical findings on brain imaging, and the characteristic clinical phenotypes of the disorder. The majority of the reviewed cases presented typical features of PTBHS disease as discussed above [4,5].

Many of these cases presented mainly with ophthalmological manifestations, including high myopia and retinal dystrophy. Of these cases, a two-and-a-half-year-old girl showed mild bilateral posterior subcapsular cataract (PSC), severe retinal thinning temporally, fundi with myopia, and straightened blood vessels nasally and possible vitreoretinal degeneration [7]. Another case with similar ocular features is about a three-year-old boy who demonstrated a large area of non-perfusion in the temporal macula on fundus examination, thinning of the retina on optical coherence tomography (OCT), and decreased density of both the deep and superficial macular vascular plexuses on OCT angiography [8].

A worthwhile point to mention here is that there is a wide variation in the severity among the typical cases themselves according to the variant of the *LAMA1* gene mutation. A cohort study published in 2016 ended with the identification of 16 novel *LAMA1* variants among 17 patients with PTBHS from 15 unrelated families [9]. An interesting article published in 2022 discussed in depth how the severity of the ocular manifestations differs from one patient to another based on the variant of the *LAMA1* gene mutation. The study involved four patients (three siblings and one unrelated child) diagnosed with PTBHS but presented with significant variability of retinopathy. All four children presented with myopia, two of the three siblings with *LAMA1* variant, c.1492del p. (Arg498Glyfs*25) had ocular motor apraxia, and the other sibling with* LAMA1* variant, c.3065del p. (Gly1022Valfs*2) presented with severe retinopathy. The unrelated child with a *LAMA1* deletion spanning exons 17-23 presented with abnormal development of retinal vasculature that was complicated with vitreous hemorrhage and neovascular glaucoma [10].

In contrast to the typical presentations, a few cases were reported with special clinical presentations. Of these cases, a case was published in 2022 about an eight-month-old baby boy who was diagnosed with PTBHS by an antenatal ultrasound that had shown ventriculomegaly at eight months. This baby presented with hypotonia, focal seizure, developmental delay, and poor eye contact but it was unique because his postnatal brain MRI revealed supratentorial abnormalities, which have not been described in any of the previously published articles [11]. A recently published article in 2023 was about a baby girl who was confirmed with the diagnosis of PTBHS at 26 weeks of pregnancy by fetal MRI that revealed brain ventriculomegaly, but what made this case special was the presentation of esophageal atresia, which was not reported in any published cases before [6].

In our case, the diagnosis was not that easy. It relied on the clinical presentation of the patient and the physician’s experience for the most part because the brain MRI findings were not very easy to detect, unlike the previously reviewed published cases. For that, a few valuable lessons can be taken from this case. Firstly, if the child is presenting with delayed growth with suspicious clinical findings, he/she should be referred to a specialized physician as soon as possible and a genetic factor should be kept in mind. Secondly, a premarital genetic test is a very helpful screening test to help increase the chances of having a healthy baby, especially if the couples are consanguineous. Thirdly, if it happens and the baby is born with a genetic disease/syndrome, the parents should be advised to be patient, get educated about their child’s condition, and follow the instructions of their treating doctor. Being genetic, almost there will be no final cure for the disease and the treatment will be supportive. Nevertheless, following up with the specialized doctors regularly will strongly improve the prognosis of the patient, as was seen in our case.

## Conclusions

Since there is almost no definitive cure for most genetic/inherited diseases, doctors should be careful before reassuring the patient or parents by paying much attention to the small details in the clinical history, examination, as well as investigations, and making a good correlation between the clinical findings and the work-up results. Moreover, a specialized doctor should be involved if there is any small concern in any case to avoid any preventable poor outcomes.

This case highlights the importance of the multidisciplinary team for early correct diagnosis and management and advocates genetic screening in the population to reduce genetic disorders. A vital last point is to focus on publishing more articles and conducting research on PTBHS to increase awareness and improve understanding of this disease.
